# CancerSiamese: one-shot learning for predicting primary and metastatic tumor types unseen during model training

**DOI:** 10.1186/s12859-021-04157-w

**Published:** 2021-05-12

**Authors:** Milad Mostavi, Yu-Chiao Chiu, Yidong Chen, Yufei Huang

**Affiliations:** 1 Greehey Children’s Cancer Research Institute, University of Texas Health San Antonio, San Antonio, TX 78229 USA; 2 Department of Electrical and Computer Engineering, University of Texas at San Antonio, San Antonio, TX 78249 USA; 3 Department of Population Health Sciences, University of Texas Health San Antonio, San Antonio, TX 78229 USA

**Keywords:** Deep learning, One-shot learning, Genomics, Cancer type prediction, Primary and metastatic tumors, Cancer gene markers

## Abstract

**Background:**

The state-of-the-art deep learning based cancer type prediction can only predict cancer types whose samples are available during the training where the sample size is commonly large. In this paper, we consider how to utilize the existing training samples to predict cancer types unseen during the training. We hypothesize the existence of a set of type-agnostic expression representations that define the similarity/dissimilarity between samples of the same/different types and propose a novel one-shot learning model called CancerSiamese to learn this common representation. CancerSiamese accepts a pair of query and support samples (gene expression profiles) and learns the representation of similar or dissimilar cancer types through two parallel convolutional neural networks joined by a similarity function.

**Results:**

We trained CancerSiamese for cancer type prediction for primary and metastatic tumors using samples from the Cancer Genome Atlas (TCGA) and MET500. Network transfer learning was utilized to facilitate the training of the CancerSiamese models. CancerSiamese was tested for different *N*-way predictions and yielded an average accuracy improvement of 8% and 4% over the benchmark 1-Nearest Neighbor (1-NN) classifier for primary and metastatic tumors, respectively. Moreover, we applied the guided gradient saliency map and feature selection to CancerSiamese to examine 100 and 200 top marker-gene candidates for the prediction of primary and metastatic cancers, respectively. Functional analysis of these marker genes revealed several cancer related functions between primary and metastatic tumors.

**Conclusion:**

This work demonstrated, for the first time, the feasibility of predicting unseen cancer types whose samples are limited. Thus, it could inspire new and ingenious applications of one-shot and few-shot learning solutions for improving cancer diagnosis, prognostic, and our understanding of cancer.

**Supplementary Information:**

The online version contains supplementary material available at 10.1186/s12859-021-04157-w.

## Background

Cancer is a condition of abnormal cell growth. Cancer that is found in the original tissue where it is formed is called primary cancer. When cancer cells spread to nearby normal tissues, or invade distant parts of the body, they develop metastatic cancer, which is the ultimate cause of death in cancer patients [1]. Cancer is an extremely complex and heterogeneous disease, manifested in individual tumors' unique genetic makeup [2]. More than 100 different cancer types are discovered to originate from various organs and sub-tissues. Yet, the same cancer type's molecular signatures can vary with its location, stage, and ultimately patients. To gain insights into the genetic markers and molecular mechanisms of different cancers, comprehensive genomic studies such as the Cancer Genome Atlas (TCGA) [3, 4] have generated and interrogated the genetic and omics (epigenomic, transcriptomic, and proteomic) profiles from large cohorts of cancer patients with some of the most common cancer types. It becomes increasingly clear now that as much as molecular profiles can accurately predict current cancer types, the spectrum of cancer transcends existing tumor lineages, underscoring the need for a molecular-based classification of individual tumors. This emergent perspective of cancer also fosters a more effective “precision cancer therapy," which advocates specialized diagnosis and treatments based on individual patients' molecular makeup [5].

This paper considers cancer classification based on gene expression data using machine learning (ML). Thanks to efforts like TCGA, the prevailing strategy nowadays is to train a classifier using tumor samples with annotated cancer types. Earlier efforts simply classified cancer from normal samples using neural networks [6]. Although they reported good classification accuracy, the model was not designed to classify precise cancer types. Several recent studies have applied deep learning (DL) models to classify tumor samples into correct cancer types. To take advantage of image-based convolutional neural networks (CNNs), approaches proposed in[7, 8] converted 1-dimensional (1-D) gene expression of cancer samples in TCGA into 2-dimensional (2-D) image-like inputs and applied CNN to classify 33 cancer types. Following a similar idea, DeepInsight, iSOM-GSN, and REFINED [9–11] have also attempted to incorporate gene correlations into the converted 2-D image to improve further the cancer type classification accuracy of CNN. In [12], a 1D-CNN model applied directly to the 1-D gene expression data was also proposed to classify 33 TCGA cancer types plus an additional normal tissue type. It showed that the 1D-CNN could achieve comparable performance with CNNs using 2D-converted gene expression but had 100 times fewer parameters than the CNN proposed in [8]. As the training sample size is commonly small for cancer classification, this simpler 1D-CNN is favored because it has less tendency to overfit the training data and is more robust with a better ability to generalize.

Impressive performance notwithstanding, these strategies have limitations, restricting their adoption for personalized cancer classification in the era of precision oncology [13]. First, the high classification accuracy is predicated on the availability of large-scale well-annotated tumor datasets like TCGA. With the TCGA project's conclusion, it would be infeasible to duplicate TCGA’s effort for additional cancer types. Second, for rare tumors, one could never expect to collect sufficient samples necessary for training an ML model with satisfactory performance. Above all, as cancer classification quickly shifts to more refined, molecular-based characterizations, we expect to see a much larger number of cancer types. The current strategy is inept with this multitude because whenever new cancer types emerge and genome-profiling technologies are updated, the classifier needs to be completely re-trained before it can be applied again.

**Fig. 1:**
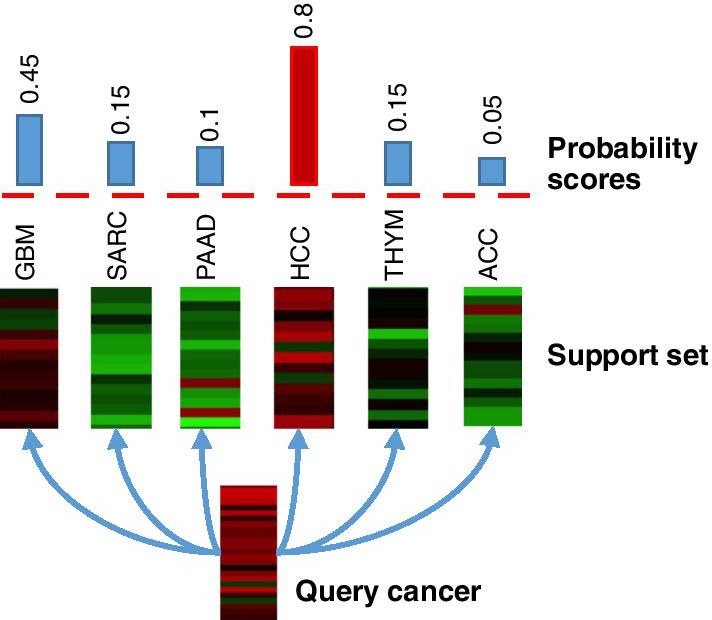
Illustration of a 6-way one-shot prediction of cancer types for a query tumor sample. A query sample is compared with each sample in a support set, which includes 6 samples, each from a cancer type. A machine learning algorithm computes the probability of similarity between the query and every support sample. The final predicted type (HCC) is the sample type in the support set that has the highest probability of being similar to the query sample

Recognizing these limitations of the current strategy, we consider the problem of utilizing samples from cancer types of existing collections such as TCGA to build and train a model to predict new cancer types unseen during the training but with limited samples. To this end, we propose a novel one-shot learning strategy, where we only require a single “support” sample to be collected from each new cancer type, a drastically reduced requirement from typical TCGA collection with ~ 500 samples per cancer type. Cancer classification of a query sample is carried out by comparing it against a set of support samples, one for each cancer type, a classic one-shot learning task (Fig. 1). One-shot learning was first proposed in computer vision for tackling data scarcity in applications such as personal identification [14, 15]. A particular class of one-shot learning models known as Siamese convolutional neural networks (SCNNs) has been shown to be a powerful similarity metric learning model and has been applied for addressing a variety of bioinformatics and medical imaging problems including drug response similarity prediction by ReSimNet [16], sequence embedding and alignment by SENSE [17], cell types identifications by MapCell [18], protein–protein interaction prediction [19], representation learning of proteins by TriplotProt [20], and representation learning of medical images [21]. With these studies' successes, we hypothesize that there is a set of type-agnostic marker genes whose expression profiles define the similarity/dissimilarity between samples of the same/different cancer types. Therefore, we shift our attention from predicting the cancer type of the query sample to predicting similarity vs. dissimilarity between a pair of query and support samples. This new perspective enables us to train an SCNN model with paired samples from the same or different cancer types as replicates for label “similar” or “dissimilar”, respectively, thus significantly reducing the need for collecting large samples for each cancer type. This new strategy advocates sampling more types as opposed to sampling more tumors of the same type, a new practice in line with the nature of precision oncology. Lastly, because the maker genes generalize across cancer types, the trained model can be directly applied to classify new cancer types that the model has not seen in the training data.

To test this hypothesis, we developed the CancerSiamese, an SCNN model that contains two identical 1D-CNNs, which learn cancer type representations of query and support samples, followed by a metric-learning layer to predict if the representations from the query and support sample are similar or not. We trained and tested CancerSiamese on samples from 29 primary and 20 metastatic cancer types to predict unseen primary and/or metastatic tumor types and conducted comprehensive investigations of the marker genes learned by CancerSiamese. Our work is noticeably different from AffinityNet [22], a recently developed kNN-based graph attention few-shot learning model, which aimed to address data scarcity and was also applied for cancer type prediction. AffinityNet is not a one-shot learning model and thus needs more than a single sample for prediction. Also, AffinityNet is very limited in its scope for cancer type predictions. First, it was trained for only two primary cancer types. Second, similar to all other existing cancer classification approaches, it was trained to predict only the cancer types in training, so it could not predict novel cancer types. Finally, AffinityNet did not attempt to interpret its predictions and thus did not inform markers and functions underlying the prediction.

The organization of the rest of the paper is as follows. In Methods, we describe the datasets and preprocessing steps necessary for one-shot learning training. The architecture of CancerSiamese network and network-transfer learning, and the gradient-based interpretation method that we have adopted to extract the marker genes of CancerSiamese were discussed in this section. In Results, the performances of CancerSiamese in predicting unseen primary and metastatic tumors were examined. The model interpretation was performed to uncover marker genes that potentially explain cancer types learned by CancerSiamese.

## Results

### Model training

**Fig. 2:**
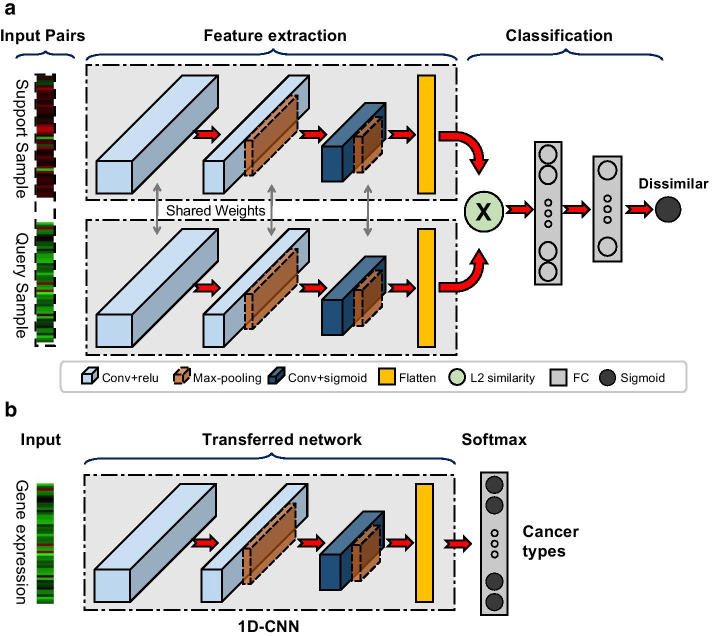
Architecture and network transfer training scheme for CancerSiamese. **a** CancerSiamese model architecture. **b** The architecture of 1D-CNN, which is pre-trained for cancer classification to initialize the feature extraction part of CancerSiamese

CancerSiamese takes gene expressions from a query sample and a support sample as input and outputs the probability that the query is from the same cancer type as the support. Its architecture is inspired by the Siamese network [23] (Fig. 2a; see the Methods section for architectural details). CancerSiamese networks were trained on TCGA and MET500 (refer to the Methods section for details) training datasets separately with Keras DL platform with the Tensorflow backend [24]. To assess the relationship between the number of training cancer types and prediction performance, three CancerSiamese networks for primary cancer prediction were trained using three different sets of primary cancer types, where the total number of cancer types was $9, 14,$ or $19,$ respectively (refer to the Methods section). In contrast, only one CancerSiamese network was trained for metastatic tumor prediction, using samples from 10 metastatic cancer types. Network transfer learning was conducted from the training of each CancerSiamese model, where the initial weights of the 1D-CNN feature extractors (Fig. 2a) were set as those in the 1D-CNN for classification of cancer types pretrained on the same training set (Fig. 2b). For example, the CancerSiamese model trained with 19 primary cancer types took the weights of the 1D-CNN classifier for classifying the same 19 primary cancer classes (Fig. 2b). The weights for the rest of the layers (i.e. FC and sigmoid) were initialized by Xavier Initialization as suggested by [23]. Each CancerSiamese network was optimized with a binary cross-entropy loss and trained with 20,000 training iterations, where each iteration includes a batch of 128 pairs with an equal number of matched and mismatched pairs, all chosen randomly from the corresponding training dataset (Table 1). The network parameters were optimized by Adam optimizer and all of the hyperparameters were tuned manually and summarized in Additional file 1: Table S1 and Table S2.

**Table 1:** Number of samples for different training and test datasets

	# of training cancer classes	# of training samples	Total # of test samples for 20,000 one-shot task
TCGA	9	1990	1895
	14	6180	
	19	8445	
MET	10	516	249

The trained CancerSiamese networks were tested for different *N*-way predictions (*N* = 6, 8, and 10). For an *N*-way prediction, CancerSiamese compared a query sample with a support set of *N* samples, each from a different cancer type. The type of the query sample was predicted as the type of the paired support sample if the pair received the highest probability by CancerSiamese out of the *N* pairs. The prediction was counted as correct if the predicted type was the same as the true type of the query sample. For every *N*-way prediction, we tested CancerSiamese on 20,000 randomly selected query samples and the corresponding support set from the test dataset and each support set contained *N* randomly selected samples, each from a different cancer type but one of them coming from the same cancer type as the query sample. For example, Fig. 1 depicts a 6-way prediction task, where six random cancer types are selected from the test set as the support set and a random sample (HCC in Fig. 1) from one of six cancer types is chosen as the query. The accuracy performance of 6-way prediction is calculated as the number of correct predictions out of 20,000 6-way predictions. The total number of samples and classes for each training and test datasets are presented in Table 1. All of the codes can be found at https://github.com/MMostavi/CancerSiamese.

### Predicting types of unseen primary and metastatic tumors with a single support sample from each class

We evaluated CancerSiamese networks' performance for predicting the type of a query sample from certain primary and metastatic cancer types that were unseen during training. We investigated the impact of training sets with different types and numbers of *N*-way predictions (*N* = 6, 8, and 10) on the prediction performance. For each performance, we computed the prediction accuracy based on 20,000 testing trials. The 1-NN classifier's accuracy was also computed as the baseline performance; 1-NN is widely used as a benchmark model for comparing the performance of few-shot learning models. For an *N*-way prediction, 1-NN calculates the Euclidean distance between the query and a support samples' gene expression and selects the label that has the minimum distance to the query sample as the predicted type.

**Fig. 3:**
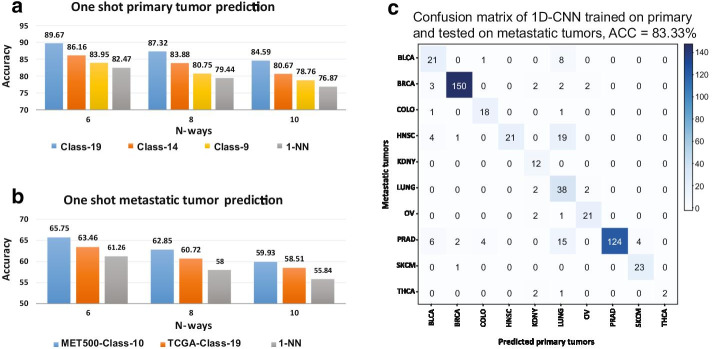
Performance of CancerSiamese for predicting unseen primary and metastatic cancer types. **a** Accuracies for predicting primary tumor types. **b** Accuracies for predicting metastatic tumor types. **c** Confusion matrix of 1D-CNN for cancer type classification trained on 10 primary cancer types and tested on corresponding 10 metastatic tumors in the training set

As seen in Fig. 3a, for all three different numbers of primary training cancer types, CancerSiamese outperformed 1-NN for all three different *N*-way predictions. Particularly, the model trained with 19 types achieved the highest performance with 89.67%, 87.32%, and 84.59% accuracy for 6, 8, and 10-way predictions in test samples, respectively. They also represent 7–8% improvement margin over 1-NN. Since the training and testing sets contain disjoint cancer types, these high accuracies suggest that there are discriminative gene expression markers shared among all cancer types that CancerSiamese models have successfully learned from training data and predicting testing samples using these markers. Besides, we also observed that increasing the number of training types from 9 to 19 increased prediction accuracy. This suggests that by adding more cancer types to the CancerSiamese training, CancerSiamese improved the learned representation of similar and dissimilar cancer types. Therefore, the richer representation of 19 primary cancer types could help improve the model's generalization to have a better prediction on unseen cancer types. In other words, the diversity of the training samples leads to improved accuracy.

CancerSiamese was further trained on the 10 cancer types of metastatic training samples (MET500). The accuracies for different *N*-way predictions of metastatic tumors (MET500 testing samples) are shown in Fig. 3b. The model's accuracy was compared to the model trained with 19 cancer types from TCGA samples (orange color bars) and 1-NN (gray color bars). We observe that CancerSimaese with 10 metastatic types achieved the best performance with approximately 4—5% accuracy improvement in all different *N*-way predictions compared with 1-NN. However, the accuracies are in the low 60% indicating that expression signatures that define similar/dissimilar metastatic types learned from 10 training classes may not yet be fully generalized well to discriminate the signatures in testing samples. This could be partially due to the smaller metastatic training samples and also potentially higher expression heterogeneity in metastatic tumors, or lower sample diversity in training.

Interestingly, the accuracies of CancerSiamese trained with 19 primary tumor types alone (65.75%) are about 2% accuracy reduction of those by CancerSiamese trained with metastatic cancer samples (63.46%), suggesting that the marker expression signatures learned from the 19 primary cancer types share significant similarity with those learned from the 10 metastatic cancer types. In fact, the 10 metastatic cancer types overlap with 10 of the 19 primary cancer types. To further verify if the 10 overlapping primary and metastatic cancer types have shared expression signatures, we trained a 1D-CNN using the training samples from these 10 primary cancer types and tested it on the training samples of the 10 metastatic cancer types. As expected, this 1D-CNN model could predict the metastatic tumors in MET500 with an accuracy of 83.33% (Fig. 3c). The confusion matrix of this experiment (Fig. 3c) further delineates the shared gene expression signatures between primary and metastatic tumors, agreed with earlier studies that have also shown majority genes’ expression of primary and metastasized tumors resemble each other [25–27]. Finally, CancerSiamese trained with either 19 types of primary tumors or metastatic tumor samples outperformed 1-NN (Fig. 3b), demonstrating once again the ability of CancerSiamese in learning similarity/dissimilarity between cancer types.

### Identification and analysis of marker genes for the primary cancers learned by CancerSiamese

**Fig. 4:**
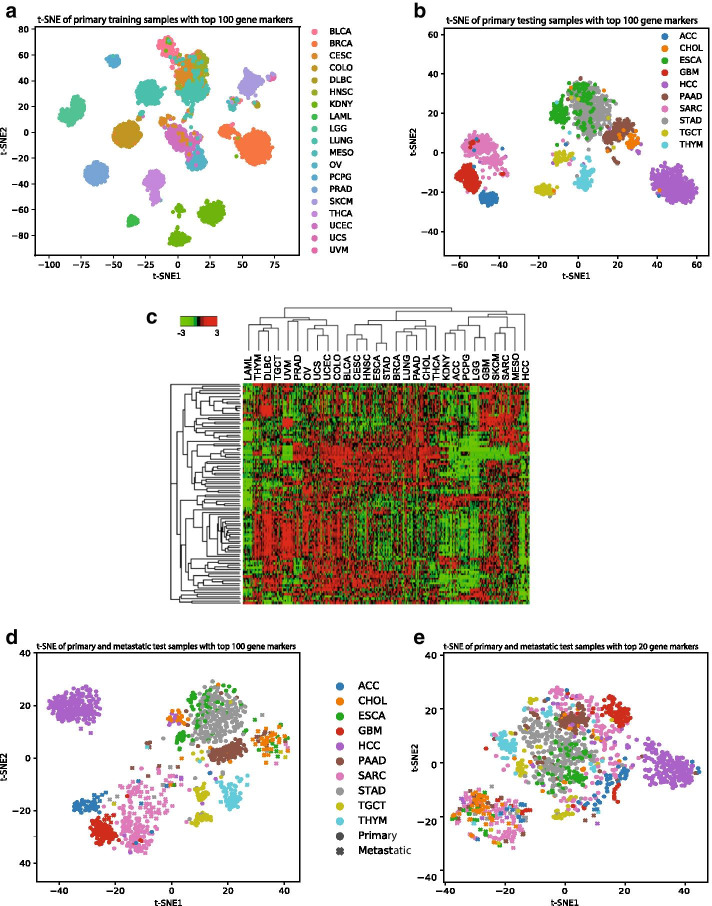
t-SNE plots and heatmap of primary and metastatic test samples with top gene markers. **a** t-SNE of primary training samples with top 100 gene markers. **b** t-SNE of primary testing samples with top 100 gene markers. **c** Heatmap of the top 100 marker genes across all 29 primary cancer types. Cancers were clustered by the average per cancer type. Gene expression levels were *z*-transformed per gene. **d** t-SNE of primary and metastatic test samples together with the top 100 gene markers. **e** t-SNE of primary and metastatic test samples together with the top 20 gene markers

In the previous section, we pointed out that the good performance in predicting primary samples of unseen classes implies that CancerSiamese learned unique expression markers, which are shared among all cancer types between disjoint training and testing sets. Because the CancerSiamese model trained on 19 primary cancer types had the best accuracy, we first selected it to investigate how CancerSiamese makes the prediction and uncover the marker genes learned by CancerSiamese. To this end, we randomly selected up to 3000 unique pairs of expressions from the same type for each of the 19 primary cancer types and used GBSM to calculate $\overline{{\varvec{w}} }$ (See Methods), whose elements represent the corresponding genes’ score to be marker genes (the ranked list of genes and scores can be found in Additional file 2). To determine the marker gene set, we performed a stepwise greedy forward selection [28], a popular feature selection method. Specifically, we ranked the genes in decreasing order based on their corresponding score. Next, we performed 1-NN for 6-way prediction on the test samples using multiple of 10 genes in the ranked list from the top (Table 2). The best accuracy was achieved with the top 100 genes, where the accuracy (82.38%) is virtually the same as that obtained from using all genes (82.47%, Fig. 3a). Therefore, we selected the top 100 genes as the marker genes (Additional file 2). Examining the t-SNE plots of training and testing samples using these marker genes further confirmed their discriminative power (Fig. 4a, b) as samples from the same type are mostly grouped in clearly separated clusters. The heatmap of the markers demonstrated cancer-type specific expression patterns (FIG. 4c). To investigate the functions of identified marker genes, we utilized The Database for Annotation, Visualization and Integrated Discovery (DAVID, v6.7) [29, 30] to search for Gene Ontology (GO) terms of molecular functions and biological processes, and KEGG and Biocarta pathways. To ensure a meaningful functional annotation analysis, we slightly relaxed the criterion and analyzed the top 5% (243 genes) of the ranked gene significant vector yielded from CancerSiamese. As shown in Additional file 1: Table S3, the top functional annotation clusters included translation, apoptosis, cell growth, response to oxidative stress, extracellular matrix (ECM), and response to wounding. Most of these functions play essential roles in cancer. Apoptosis and cell growth are key determinants of cancer cell proliferation [31]. ECM and wound healing are critical components/indicators for the migration and metastasis of tumors and represent the fundamentally different tumor microenvironment between solid and hematopoietic cancers. The oxidative stress is known to trigger tumor progression and modulate chemotherapies' response [32, 33]. Taken together, the top marker genes capture critical functions executed by tumors to survive and metastasize, thus potentially making the differences between tumors and between primary and metastatic tumors.

**Table 2:** Accuracies of the stepwise greedy forward selections for selecting marker genes for primary cancers (column 2) and their ability for predicting metastatic cancer types (column 3) and discriminating primary from metastatic cancers (column 4)

Number of top genes	6-way prediction (primary cancers; 1-NN)	6-way prediction (metastatic cancers; 1-NN)	Classification of primary and metastatic cancers (GNB)
10	78.64	37.4	88.35
20	80.74	38.11	*95.22*
30	81.61	37.58	92.87
40	81.8	38.12	93.13
50	81.8	37.29	93.55
100	*82.38*	37.51	93.28
150	82.23	37.08	94.38
200	82.1	37.36	95.1

### Identification of metastatic tumor marker genes learned by CancerSiamese and their relationship with those for primary tumors

Because this CancerSiamese model was also shown to achieve 63.46% accuracy when being extrapolated to the 6-way prediction for metastatic unseen sample types, we first wondered if these marker genes might also be responsible for this accuracy in predicting similar metastatic cancer types. To this end, we performed 1-NN for 6-way prediction on the metastatic samples again using multiple of 10 genes in the ranked list from the top and the accuracies stayed between 37% and 39% (Table 2), which is considerably lower than 63.46%. This result indicates that these 100 marker genes possess predominantly similar but distinct expression patterns among samples from the same primary cancer types but not the metastatic cancer types. The t-SNE plot of the testing primary and metastatic samples (Fig. 4d) further confirmed this finding. Although many studies have shown that metastasized tumors are similar to the parent primary tumors, many others have also pointed out the increased genome heterogeneity in metastasized tumors due to their different tumor microenvironment and immunological conditions [34, 35]. Our result suggests that the marker genes that define similar/dissimilar metastatic cancer types are different from those of primary cancer types. Therefore, we hypothesized that the markers that explain the unique characteristics of metastasized tumors might also serve to discriminate two metastatic cancer types. Interestingly, we observed that metastatic samples of 6 types (PAAD, STAD, ESCA, CHOL, SARC, and ACC) were separated from their corresponding primary cancers (Fig. 4d). This further implies that these 100 marker genes also carry discriminative expression patterns that can be used to separate metastatic from primary samples, for at least these 6 cancer types. To validate this observation and determine the specific subset of the markers responsible for this discriminative power, we performed the stepwise greedy forward selection on these 100 genes. For each increment of 10 genes in the ranked list, we trained a Gaussian Naïve Bayes (GNB) classifier to classify primary and metastatic cancer using the testing samples and determine that the top 20 genes were the discriminative markers (Table 2). The t-SNE plot of test samples using these 20 genes (Fig. 4e) showed a clear separation between primary and metastatic cancer samples than using 100 markers (Fig. 4d). We noted that many of the 20 genes were well-known to be strongly associated with metastatic mechanisms in many cancer types, such as *TAGLN2* [36], *S100A11* [37, 38], *CD74* [39], *TMSB10* [40, 41], and *ALDOA* [42].

To further determine the marker genes for metastatic cancers, we performed a stepwise greedy forward selection on the CancerSiamese trained on the metastatic tumors with 10 classes and identified 250 marker genes (Additional file 2), which produced an accuracy of 60.05% by 1-NN (Additional file 1: Table S4), which is very close to the accuracy by 1-NN from all genes (61.26%, Fig. 3b). Comparing these metastatic marker genes with 100 markers for primary cancers yielded little overlapping (only 24 common genes), demonstrating the clear difference between metastatic and primary markers again. These different genes in the metastatic markers list are responsible for the additional improvement over the 38.12% accuracy achieved by primary markers (Table 2). Functional annotation analysis of these 250 gene markers confirmed an association with cell–cell adhesion while also highlighted other fundamental molecular functions, such as nucleotide binding and peptide modification (Additional file 1: Table S5).

## Discussion and conclusion

In this paper, we proposed CancerSiamese, a one-shot learning model for predicting primary and metastatic cancer type of a query expression sample based on a set of support expression samples, one from each cancer type. This model was developed based on the hypothesis that there exists a set of marker genes whose expressions define the similarity/dissimilarity between samples of the same/different types. CancerSiamese includes two parallel 1D-CNN with shared weights for extracting expression representations from the input query and support samples and a metrics learning network to assess the similarity between the two representations. This model was trained for primary and metastatic tumors separately and tested for different *N*-way predictions. The test results showed high prediction accuracy for primary cancers (89.67%, 87.32%, and 84.59% for 6, 8, and 10-way predictions), representing about 7–8% improvement over 1-NN. We also showed that these prediction accuracies could be further improved by including more cancer classes in the training data. Interestingly, because the testing was done on cancer types unseen during training, these markers learned by CancerSiamese from the training cancer types seem to be cancer type agnostic, i.e., they are also the markers for the independent test cancer types. To determine this list of marker genes, we applied GBSM coupled with the stepwise greedy forward feature selection algorithm to interrogate CancerSiamese, which resulted in 100 maker genes capable of predicting cancer types with equivalent accuracy (Table 2). t-SNE plots further verified the cancer-type agnostic nature of these marker genes. Further analysis also showed that these maker genes are unique to the primary cancer types and the top 20 of them possess significant discriminative power for discerning primary from metastatic cancers. Functional enrichment analysis of these marker genes revealed their association with many important functions such as translation, apoptosis, cell growth, response to oxidative stress, extracellular matrix (ECM), and response to wounding were observed.

**Fig. 5:**
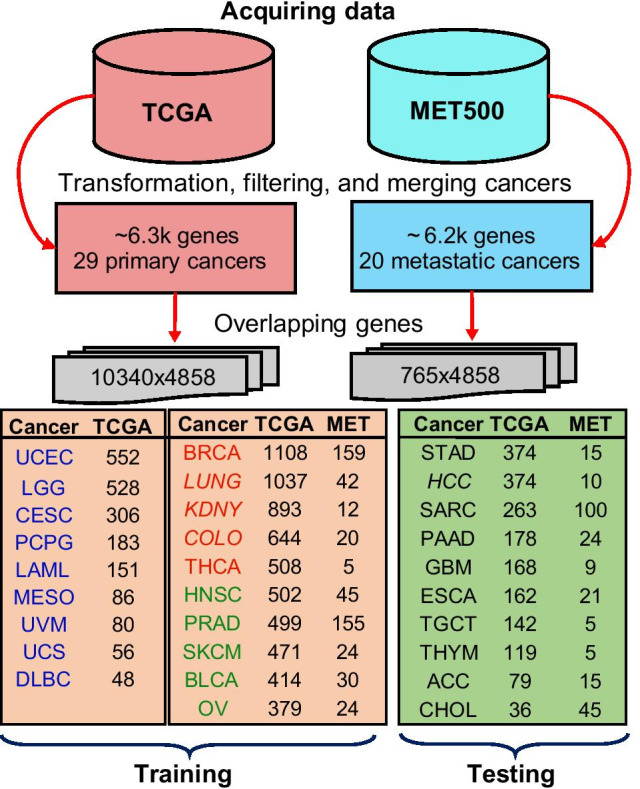
Preprocessing workflow for extracting the training and testing primary and metastatic tumor samples. After downloading the TCGA and MET500 datasets, data preprocessing, including filtering genes and merging related cancer types, was performed. 4,858 common genes in TCGA and MET500 were retained and then samples were divided into training and testing sets according to the number of primary cancer types in TCGA. Cancer types in italic font are merged tumor groups, as described in the Methods section. Three training datasets for primary cancers were created and included 9 (blue labeled), 14 (blue + red labeled), and all 19 cancer types, respectively

In contrast, the CancerSiamese trained for predicting metastatic cancer acquired accuracies only in low 60% during testing, even though they still outperformed 1-NN by about 4%. Given that the number of MET500 samples for each cancer type is 10- to 20-fold less than those in TCGA (refer to Methods section Fig. 5), these low accuracy could be improved with an expanded collection of metastatic tumor types as witnessed in Fig. 3a for the primary cancers. Nevertheless, metastatic tumors with the same tissue of origin could exhibit disparate genomic features in different metastatic sites. This heterogeneity compounded by the impurity of metastatic tumor samples could further contribute to the low prediction accuracies. A refined classification of metastatic cancer types that also consider metastatic sites could help improve the performance. However, this also requires collecting more metastatic tumor samples. Despite the low performance, we still identified 200 marker genes, which account for 90% of the CancerSiamese’s performance. However, we observed a little overlap with the marker genes for the primary cancers.

These results, especially those for primary cancers, serve to validate our hypothesis and, for the first time, demonstrated the possibility of applying one-shot learning for expression-based cancer type prediction. This new paradigm of one-shot learning recognizes the reality of having very few samples for each tumor type in the era of precision oncology. It provides a principled approach to meet the need for data-driven cancer diagnosis with small samples. Extension of CancerSiamese into a more versatile few-shot learning model will directly impact the practical application of CancerSiamese for precision tumor diagnosis. As the small sample size is one of the key machine learning challenges in precision oncology and general precision medicine research, this work could inspire new and ingenious development of one-shot and few-shot learning solutions to improve cancer therapy our understanding of cancer.

## Methods

### Dataset

RNA-Seq data from TCGA and Integrative Clinical Genomics of Metastatic Cancer known as MET500 [4] were downloaded by R/Bioconductor package TCGAbiolinks (https://www.bioconductor.org) and UCSC Xena (https://xenabrowser.net/datapages/) in December 2018, respectively. In total, TCGA contains 10,340 samples from 33 different primary cancer types, whereas MET500 includes 765 samples from 20 cancer types of metastatic tumors. Additional file 1: Table S6 shows the 11 different metastatic tumor types in MET500. The gene expressions of these two datasets were transformed by log2 (FPKM + 1) where FPKM (fragments per kilobase per million mapped reads) is the unit of gene expression level.

We used the tissue of origin to label both primary and metastatic tumors. As the first preprocessing step, all primary cancer types with a similar origin were grouped together and then renamed with their counterpart metastatic tumor label in MET500. Specifically, four primary cancer groups in TCGA known as [(COAD, READ), (KIRP, KIRC, KICH), (LUAD, LUSC), LIHC] were renamed as their corresponding metastatic labels in MET500 [COLO, KDNY, LUNG, HCC], respectively. After this process, the number of primary cancer types in TCGA dropped from 33 to 29 (i.e. four groups with similar origin were merged). Out of 29 types, 9 are unique to TCGA (primary) cancers, whereas 20 are common between TCGA and MET500 (primary and metastatic) cancer types. See Fig. 5 for the names and sample sizes of each cancer type.

Similar to the processing strategy as in [12, 43, 44], genes with both mean and standard deviation of less than 0.8 across all samples, regardless of their cancer types, in TCGA and MET500 were filtered out. This preprocessing step was designed to reduce the effect of noise as well as genes that have little discriminative value in these two datasets. As shown in Fig. 5, this step reduced the number of genes in TCGA and MET500 to around 6.3 K and 6.2 K, respectively, and 4,858 common genes in both datasets were determined. These 4858 genes were kept for all primary and metastatic tumors in TCGA and MET500, respectively.

The training data include samples from 9 unique primary cancer types and 10 additional cancer types with primary and metastatic samples. The training set were selected based on their higher number of primary samples or when only TCGA samples were available (Fig. 5, brown color). The testing data include the primary and metastatic samples from the remaining 10 cancer types (Fig. 5, green color). It is important to note that the testing set does not share any common cancer types with the training set. This is because our goal is to predict query samples from cancer types *unseen* in the training set.

### Proposed network model for CancerSiamese

We consider a scenario where we have a query and *N* support gene expression samples, each from a different cancer type. Among the *N* support samples, one has the same cancer type as the query sample. The goal is to determine which one of *N* cancer types the query sample belongs to, or make an *N*-way prediction. To achieve the goal, we propose CancerSiamese, a one-shot learning model inspired by the Siamese network [23], that takes a pair of query and support samples, using DL model to extract appropriate data representation for both query and support, and then computes the probability that the query is from the same cancer type as the support. After we apply CancerSiamese to all the samples in the support set, the query sample's predicted cancer type is taken as the one with the highest probability (Fig. 1).

CancerSiamese consists of two identical CNNs applied to the query and support sample individually, followed by a similarity metric network (Fig. 2a). We adopted the 1D-CNN proposed in [12], due to its simplicity and high accuracy for TCGA cancer type prediction, to learn a low-dimensional representation of tumor gene expression. In particular, after the first 1D convolution layer, we added two consecutive 1D convolution and maxpooling layers, and finally a flatten layer. Similar to [23], the relu activation function was selected for the first two 1D convolution layers and sigmoid for the last 1D convolution layer. For the similarity metric network, an element-wise ${L}_{2}$ distance was applied to the two feature vectors generated by the 1D-CNNs and the output was passed onto two consecutive fully connected (FC) layers followed by a sigmoid node to determine whether the input pairs belong to the same cancer type.

### Network transfer learning

Due to the large network size of CancerSiamese, training the network from scratch suffered from training instability and poor convergence, eventually resulting in less robust prediction [45, 46]. To address these training challenges, we adopted a transfer learning scheme to build the CancerSiamese model. Specifically, we first trained a 1D-CNN using the cancer training samples from TCGA for cancer type classification. Afterward, we removed the classification layer and took the remaining trained 1D-CNN for CancerSiamese (Fig. 2b). During the training of the CancerSiamese model, these 1D-CNN networks were further optimized for predicting the similarity of the input samples. Because the 1D-CNN has already been trained to extract expression representations important for cancer type classification, the weights are closer to the one-shot learning optimal. Therefore, initializing from this pretrained 1D-CNN makes CancerSiamese training more stable with faster convergence.

### Identifying the marker genes from CancerSiamese

We hypothesized that the CancerSiamese trained on primary and metastatic tumors (the details are described in the Results section) rely on marker genes to define a similar cancer type. To uncover the marker genes, a deep learning interpretation approach known as Guided Backpropagation Saliency Maps (GBSM) was employed [47] to compute each gene score to be a potential marker gene. GBSM computes such scores for every gene in pair of gene expression samples by backpropagating a gradient from the model's output to the input to examine the impact of input genes on the model’s final decision. We have previously applied this approach to extract significant cancer markers across different primary cancer types [12]. Briefly, for an input pair of expression samples ${{\varvec{x}}}_{0}$ and ${{\varvec{x}}}_{1}$ **(**vectors of length 4858 in our CancerSiamese model**)** from the same cancer type, GBSM was applied to compute the corresponding gradient vectors ${{\varvec{w}}}_{0}$ and ${{\varvec{w}}}_{1}$, which have the the same dimension as ${{\varvec{x}}}_{0}$ and ${{\varvec{x}}}_{1}$ and whose elements represent the significance of the corresponding genes. We calculated position-wise average ${{\varvec{w}}}_{{\varvec{a}}{\varvec{v}}{\varvec{g}}}={({\varvec{w}}}_{0}+{{\varvec{w}}}_{1})/2$ to obtain a single significance vector for every pair of expressions from the same cancer type and then computed $\overline{{\varvec{w}} }$**,** averaged ${{\varvec{w}}}_{{\varvec{a}}{\varvec{v}}{\varvec{g}}}$ over all the similar pairs used for identifying marker genes, as the significant scores for each gene to be marker gene. We ranked the genes based on their score and then adopted a popular feature selection method, a stepwise greedy forward selection[28], to search for a subset of genes that produced the best classification performance. This subset is determined as the marker genes. We chose a search step size of 10 genes and applied 1-NN as the one-shot classifier for the search.

## Supplementary Information

**Additional file 1**. This file contains all of the supplementary tables from data preprocessing, model hyperparameter tuning, and interpretation of models.

**Additional file 2**. This file contains gene significant scores for each gene in primary and metastatic tumors based on CancerSiamese interpretation.

## Data Availability

The TCGA and MET500 datasets used for training CancerSiamese are publicly available at the GDC Data Portal (https://portal.gdc.cancer.gov/) and UCSC Xena platform (http://xena.ucsc.edu/), respectively, and CancerSiamese codes can be found at https://github.com/MMostavi/CancerSiamese.

## Abbreviations

MET500: MET500 metastatic cancer cohort
MET: Metastatic tumors
DL: Deep learning
ML: Machine learning
TCGA: Cancer Genome Atlas
CNNs: Convolutional neural networks
1-D: 1-Dimensional
2-D: 2-Dimensional
SCNNs: Siamese convolutional neural networks
FC: Fully connected
GBSM: Guided backpropagation saliency maps
1-NN: 1-Nearest neighbor
GO: Gene Ontology
DAVID: Database for Annotation, Visualization and Integrated Discovery
ECM: Extracellular matrix
ACC: Adrenocortical cancer
BLCA: Bladder urothelial carcinoma
BRCA: Breast invasive carcinoma
CESC: Cervical and endocervical cancer
CHOL: Cholangiocarcinoma
COAD: Colon adenocarcinoma
CNN: Convolutional neural network
DLBC: Diffuse large B-cell lymphoma
ESCA: Esophageal carcinoma
GBM: Glioblastoma multiforme
HNSC: Head and neck squamous cell carcinoma
KICH: Kidney chromophobe
KIRC: Kidney clear cell carcinoma
KIRP: Kidney papillary cell carcinoma
LAML: Acute myeloid leukemia
LGG: Lower grade glioma
LIHC: Liver hepatocellular carcinoma
LUAD: Lung adenocarcinoma
LUSC: Lung squamous cell carcinoma
MESO: Mesothelioma
OV: Ovarian serous cystadenocarcinoma
*P*: *P* Value
PAAD: Pancreatic adenocarcinoma
PCPG: Pheochromocytoma and paraganglioma
PRAD: Prostate adenocarcinoma
READ: Rectum adenocarcinoma
SARC: Sarcoma
SKCM: Skin cutaneous melanoma
STAD: Stomach adenocarcinoma
TCGA: The Cancer Genome Atlas
TGCT: Testicular germ cell tumor
THCA: Thyroid carcinoma
THYM: Thymoma
UCEC: Uterine corpus endometrioid carcinoma
UCS: Uterine carcinosarcoma
UVM: Uveal melanoma
