# Supplementary material for: CancerSiamese: one-shot learning for predicting primary and metastatic tumor types unseen during model training
Source: BMC Bioinformatics. 2021 May 12;22:244. doi: 10.1186/s12859-021-04157-w (PMC8117642; doi:10.1186/s12859-021-04157-w)
Supplement: Supplementary file 1 — Additional file 1. This file contains all of the supplementary tables from data preprocessing, model hyperparameter tuning, and interpretation of models. [file 12859_2021_4157_MOESM1_ESM.docx]

**Additional file 1.**

**Table 1**: hyperparameters of CancerSiamese

| **Layer name** | **Input** |
| --- | --- |
| Input layer | Y_0_ and Y_1_ |
|  |  |
| 1D conv | **Kernel size** = 50  **Stride** = 50  **Number of kernels** = 256  **Activation** = Relu  **Padding** = same  **Weights** = pre-trained |
| 1D conv | **Kernel size** = 10  **Stride** = 1  **Number of kernels** = 128  **Activation** = Relu  **Padding** = same  **Weights** = pre-trained |
| 1D Maxpooling | **Pool size** = 2 |
| 1D conv | **Kernel size** = 5  **Stride** = 1  **Number of kernels** = 128  **Activation** = sigmoid  **Padding** = same  **Weights** = pre-trained |
| Maxpooling | **Pool size** = 2 |
| flatten | ---- |
| L_2_ layer | ---- |
| FC | **Number of nodes** = 512  **Activation** = Relu |
| Dropout | **Dropout rate** = 0.25 |
| Output node | One node  **Activation**= sigmoid |

**Table 2**: Optimization parameters for CancerSiamese

| **Name of parameter** | **Value** |
| --- | --- |
| Learning rate | 0.000005 |
| Batch size | 128 |
| Number of training iteration | 20,000 |

**Table 3:** Top 6 functional annotation clusters enriched in the top 5% of marker genes of primary samples

| **Category^a^** | **Term** | **Number of marker genes** | **Percentage of marker genes** | ***P*-value^b^** |
| --- | --- | --- | --- | --- |
| Cluster 1 (enrichment score: 13.7) | | | | |
| MF | GO:0005198~structural molecule activity | 41 | 17.6 | 2.68E-15 |
| BP | GO:0006412~translation | 27 | 11.6 | 9.65E-12 |
| BP | GO:0006414~translational elongation | 25 | 10.7 | 3.58E-22 |
| Cluster 2 (enrichment score: 3.3) | | | | |
| BP | GO:0042981~regulation of apoptosis | 31 | 13.3 | 6.83E-6 |
| BP | GO:0043067~regulation of programmed cell death | 31 | 13.3 | 8.30E-6 |
| BP | GO:0010941~regulation of cell death | 31 | 13.3 | 8.92E-6 |
| Cluster 3 (enrichment score: 2.8) | | | | |
| BP | GO:0040008~regulation of growth | 11 | 4.7 | 4.04E-2 |
| MF | GO:0019838~growth factor binding | 10 | 4.3 | 2.38E-5 |
| BP | GO:0001558~regulation of cell growth | 9 | 3.9 | 1.08E-2 |
| Cluster 4 (enrichment score: 2.7) | | | | |
| BP | GO:0010035~response to inorganic substance | 15 | 6.4 | 3.78E-6 |
| BP | GO:0006979~response to oxidative stress | 14 | 6.0 | 1.63E-6 |
| BP | GO:0000302~response to reactive oxygen species | 8 | 3.4 | 1.60E-4 |
| Cluster 5 (enrichment score: 2.5) | | | | |
| BP | GO:0043062~extracellular structure organization | 9 | 3.9 | 3.92E-3 |
| BP | GO:0030198~extracellular matrix organization | 8 | 3.4 | 1.17E-3 |
| MF | GO:0005201~extracellular matrix structural constituent | 6 | 2.6 | 8.71E-3 |
| Cluster 6 (enrichment score: 2.5) | | | | |
| BP | GO:0009611~response to wounding | 21 | 9.0 | 2.24E-4 |
| BP | GO:0006952~defense response | 18 | 7.7 | 1.54E-2 |
| BP | GO:0006954~inflammatory response | 12 | 5.2 | 1.26E-2 |

Each cluster is represented by the largest 3 functions.

^a^BP, biological process; MF, molecular function.

^b^Modified Fisher’s exact test *P*-value from DAVID.

**Table 4**: Accuracies of the stepwise greedy forward selections for selecting marker genes for metastatic tumor prediction

| **Number of top genes** | **6-way prediction**  **(1-NN)** |
| --- | --- |
| 20 | 45.09 |
| 60 | 55.07 |
| 100 | 56.18 |
| 150 | 57.15 |
| 200 | 59.97 |
| 250 | 60.05 |
| 300 | 59.98 |
| 400 | 59.61 |

**Table 5**: Top 6 functional annotation clusters enriched in the top 250 marker genes of metastatic samples

| **Category^a^** | **Term** | **Number of marker genes** | **Percentage of marker genes** | ***P*-value^b^** |
| --- | --- | --- | --- | --- |
| Cluster 1 (enrichment score: 2.9) | | | | |
| MF | GO:0032555~purine ribonucleotide binding | 47 | 19.2 | 1.35E-4 |
| MF | GO:0032553~ribonucleotide binding | 47 | 19.2 | 1.35E-4 |
| MF | GO:0017076~purine nucleotide binding | 47 | 19.2 | 3.73E-4 |
| Cluster 2 (enrichment score: 2.0) | | | | |
| BP | GO:0031589~cell-substrate adhesion | 8 | 3.3 | 8.0E-4 |
| KEGG | hsa04510:Focal adhesion | 8 | 3.3 | 9.6E-2 |
| BP | GO:0007160~cell-matrix adhesion | 7 | 2.9 | 2.5E-3 |
| Cluster 3 (enrichment score: 1.8) | | | | |
| MF | GO:0004713~protein tyrosine kinase activity | 8 | 3.3 | 1.1E-2 |
| BP | GO:0018108~peptidyl-tyrosine phosphorylation | 5 | 2.0 | 5.4E-3 |
| BP | GO:0018212~peptidyl-tyrosine modification | 5 | 2.0 | 6.3E-3 |
| Cluster 4 (enrichment score: 1.8) | | | | |
| BP | GO:0007010~cytoskeleton organization | 13 | 5.3 | 3.8E-2 |
| BP | GO:0030036~actin cytoskeleton organization | 10 | 4.1 | 8.6E-3 |
| BP | GO:0030029~actin filament-based process | 10 | 4.1 | 1.3E-2 |
| Cluster 5 (enrichment score: 1.5) | | | | |
| BP | GO:0006811~ion transport | 21 | 8.6 | 1.5E-2 |
| BP | GO:0030001~metal ion transport | 17 | 6.9 | 2.4E-3 |
| BP | GO:0006812~cation transport | 17 | 6.9 | 1.2E-2 |
| Cluster 6 (enrichment score: 1.4) | | | | |
| MF | GO:0030246~carbohydrate binding | 10 | 4.1 | 7.8E-2 |
| MF | GO:0005539~glycosaminoglycan binding | 7 | 2.9 | 1.7E-2 |
| MF | GO:0001871~pattern binding | 7 | 2.9 | 2.6E-2 |

Each cluster is represented by the largest 3 functions.

^a^BP, biological process; MF, molecular function; KEGG, KEGG pathway.

^b^Modified Fisher’s exact test *P*-value from DAVID.

**Table 6**: Number of metastatic tumors and their colonized tissue in MET500 dataset. Rows are number of metastatic types and columns are corresponding tissue/location that they are found. MISC (“Other Cancers” as defined by MET500) and SECR (Secretory Cancer) were removed from our analysis since they do not represent any specific tumor types defined by TCGA.

|  | **Colonized Tissues/Locations** | | | | | | | | | | |
| --- | --- | --- | --- | --- | --- | --- | --- | --- | --- | --- | --- |
|  | Adrenal | Bladder | Bone_Marrow | Brain | Breast | Cervix | Colon | Liver | Lung | Lymph_Node | Oral |
| ACC | 2 |  |  |  |  |  |  | 6 | 1 | 2 |  |
| BLCA |  | 2 | 2 |  |  |  |  | 9 |  | 10 |  |
| BRCA |  | 4 | 17 | 5 | 2 |  |  | 64 | 3 | 36 |  |
| CHOL |  |  |  | 2 |  |  |  | 33 | 2 | 4 |  |
| COLO |  |  |  |  |  |  | 2 | 7 | 2 | 3 |  |
| ESCA |  |  |  |  |  |  |  | 14 |  | 3 | 2 |
| GBM |  |  |  | 9 |  |  |  |  |  |  |  |
| HCC |  |  |  |  |  |  |  | 4 |  |  |  |
| HNSC |  |  | 2 |  |  |  |  | 12 | 9 | 10 | 1 |
| KDNY |  |  | 4 | 1 |  |  |  | 4 |  | 3 |  |
| LUNG | 2 |  |  | 2 |  |  |  | 8 | 4 | 21 |  |
| OV |  |  | 2 |  |  |  | 1 | 4 | 4 | 7 |  |
| PAAD |  |  | 1 |  |  |  |  | 14 | 5 | 2 |  |
| PRAD | 1 | 2 | 39 | 1 |  |  |  | 26 | 2 | 66 |  |
| SARC |  |  | 2 |  | 1 |  |  | 9 | 22 | 2 |  |
| SKCM |  |  |  |  | 2 |  |  |  | 1 | 6 | 2 |
| STAD |  |  |  |  |  |  |  | 6 |  |  |  |
| TGCT |  |  |  |  |  |  |  | 2 |  | 3 |  |
| THCA |  |  |  |  |  |  |  |  |  |  |  |
| THYM |  |  |  |  |  |  |  | 2 | 2 | 1 |  |
| MISC |  |  | 2 | 4 |  | 2 |  | 16 | 6 | 14 | 2 |
| SECR |  |  |  | 2 |  |  |  | 3 | 12 | 6 | 3 |
